# Salivary Prevalence of Four Oral Pathogens in Postpartum Women in Northeast Romania: An Exploratory Cross-Sectional Study

**DOI:** 10.3390/pathogens15050507

**Published:** 2026-05-08

**Authors:** Giorgio Nichitean, Elena Teona Cosovanu, Oana Bejan, Silvia Ionescu, Doina Ivanov, Costin Damian, Demetra Socolov, Mihaela Grigore, Cristina Daniela Dimitriu, Cezar Foia, Ionut Luchian, Diana Tatarciuc, Irina Draga Caruntu, Luminita Smaranda Iancu, Ramona Gabriela Ursu

**Affiliations:** 1 Grigore T. Popa University of Medicine and Pharmacy, 700115 Iasi, Romania; 2 Medical Analysis Laboratory, Clinical Hospital of Pneumology, 700115 Iași, Romania; 3 “Cuza-Vodă” Clinical Hospital of Obstetrics and Gynecology, 700038 Iasi, Romania; 4 National Institute of Public Health, Iasi Regional Center for Public Health, 700465 Iasi, Romania

**Keywords:** pregnancy, oral pathogens, salivary microbiota, dental caries, gingival bleeding, Real-Time PCR, postpartum, cross-sectional study, exploratory study, Northeast Romania

## Abstract

Background: Oral dysbiosis during pregnancy has been associated with adverse outcomes, including preterm birth, premature rupture of membranes (PROM), and low birth weight, yet oral health remains an underappreciated component of routine prenatal care. Dental caries and gingival bleeding are frequently reported during pregnancy and may remain clinically relevant in the immediate postpartum period, but their relationship with specific oral pathogens in postpartum women has been insufficiently characterised, particularly in Eastern European populations. Methods: This exploratory cross-sectional, single-centre study included 60 postpartum women recruited consecutively at “Cuza-Vodă” Clinical Hospital of Obstetrics and Gynecology, Iași, Romania, between December 2025 and February 2026. All participants completed a structured questionnaire covering obstetric history, demographic characteristics, and oral hygiene behaviours and underwent a standardised clinical oral examination by two calibrated examiners. Before study initiation, the two examiners underwent a joint calibration session based on the predefined visual oral assessment criteria used in this study and agreed on uniform recording procedures for visible dental caries, self-reported gingival bleeding during brushing, tooth mobility, and overall oral status. Saliva samples were collected after delivery. Genomic DNA was extracted using a magnetic-bead protocol and analysed by Real-Time PCR using TaqMan-based assays to detect four oral pathogens: *Porphyromonas gingivalis*, *Streptococcus mutans*, *Mycoplasma salivarium*, and *Fusobacterium nucleatum*. Results: Most participants were primiparous (55.0%) and delivered at term (≥37 weeks of gestation; 78.3%). The prevalence of pathogen detection was: *P. gingivalis* 38.3% (23/60), *S. mutans* 70.0% (42/60), *M. salivarium* 71.7% (43/60), and *F. nucleatum* 100% (60/60). Poly-microbial carriage was common: 15.0% of participants carried all three variable pathogens simultaneously (*S. mutans*, *M. salivarium*, and *P. gingivalis*), and the most frequent two-pathogen combination was *S. mutans* + *M. salivarium* (30.0%). No statistically significant associations were identified between pathogen detection and clinical or obstetric variables, consistent with limited statistical power in this small convenience sample. Conclusions: This exploratory study provides the first salivary prevalence estimates for these four oral pathogens in postpartum women in Northeast Romania. The high prevalence of poly-microbial carriage, including the novel quantitative estimate for *M. salivarium*, provides an empirical foundation for power calculations and future confirmatory research integrating standardised periodontal assessment with pregnancy outcome data.

## 1. Introduction

### 1.1. Oral Health in Pregnancy: A Public Health Concern

Oral diseases remain among the most prevalent noncommunicable conditions worldwide, with dental caries and periodontal disease accounting for the largest share of the global burden and imposing substantial costs on individuals and health systems, particularly in low- and middle-income countries [1]. Periodontal disease alone can affect up to 90% of adults and has been associated, beyond its local consequences of tissue destruction and tooth loss, with several systemic conditions, including cardiovascular disease, diabetes, pulmonary disease, and adverse pregnancy outcomes [2,3].

Pregnancy represents a period of heightened oral health vulnerability. Elevated oestrogen and progesterone levels increase gingival vascular permeability, while pregnancy-associated immunomodulation amplifies the inflammatory response to dental plaque and favours the proliferation of cariogenic and periodontopathogenic species [4,5,6,7]. These physiological shifts can worsen pre-existing oral conditions and precipitate new ones, most notably pregnancy gingivitis, which affects approximately 40% of expectant women [6]. Despite this, dental attendance and awareness of oral health risks during pregnancy remain low in many populations, and oral care is often under-represented in antenatal management [8,9]. Closing this gap requires both behavioural measures and a clearer understanding of the specific microbial exposures that link maternal oral health to gestational and neonatal outcomes.

### 1.2. Periodontal Pathogens and Adverse Pregnancy Outcomes

Periodontitis is an inflammatory disease driven by dysbiosis of the subgingival biofilm, in which a susceptible host, expansion of periodontopathogenic species, and loss of commensals contribute jointly to the destruction of periodontal tissues [10,11]. The microbial architecture of this biofilm was first formalised by Socransky et al., who identified bacterial complexes as the functional units of periodontal pathology: the red complex (*Treponema denticola*, *Porphyromonas gingivalis*, *Tannerella forsythia*) is the most strongly associated with disease, while the orange complex—which includes *Fusobacterium* species, *Prevotella*, and *Campylobacter*—acts as a bridging cluster [12]. Environmental and host factors such as stress, smoking, and diabetes accelerate this dysbiotic process [13], and periodontitis is increasingly positioned as a systemic inflammatory disease with ramifications beyond the oral cavity [3].

Among these systemic ramifications, adverse pregnancy outcomes have received growing attention [14,15,16]. Preterm birth—defined as delivery before 37 weeks and reported in 5–20% of pregnancies—remains a leading cause of neonatal morbidity and mortality [14,17]. Periodontal pathogens have been mechanistically linked to preterm birth, premature rupture of membranes (PROM), low birth weight, preeclampsia, and gestational diabetes [15,16,18]. *P. gingivalis* is the most extensively characterised: its fimbriae and gingipain proteases can modify maternal and foetal tissues, and the bacterium has been recovered from amniotic fluid and placenta in affected pregnancies [18]. *F. nucleatum*, through its FadA adhesin–VE-cadherin interaction, can translocate haematogenously to the fetoplacental unit and has been detected in the placenta and amniotic fluid in cases of preterm delivery and stillbirth [15]. The clinical manifestation of periodontitis is not a prerequisite for these organisms to exert systemic effects: their presence in saliva as part of dysbiotic oral carriage—even in the absence of formal periodontal diagnosis—is considered biologically relevant in this context, and it is salivary carriage of these pathogens, rather than clinically diagnosed periodontitis, that this study characterises.

### 1.3. Dental Caries in Pregnancy and Rationale for a Targeted Salivary Panel

Dental caries is the second major oral disease burden relevant to pregnancy, driven predominantly by *Streptococcus mutans*, a keystone cariogenic species whose ecological role has been the foundation of modern caries research for over a century [19]. A meta-analysis of 1230 pregnant and 715 non-pregnant women found that salivary calcium declines in the third trimester, phosphate falls in the second and third trimesters, and salivary *S. mutans* counts increase in the latter half of pregnancy—changes which may elevate future caries risk and support the case for early screening [20]. High maternal salivary levels of *S. mutans* are a strong predictor of early-childhood caries in the offspring, establishing vertical transmission of cariogenic microbiota as an additional clinical rationale for characterising maternal oral microbial carriage at delivery and in the immediate postpartum period [21].

*Mycoplasma salivarium*, an oral commensal with opportunistic potential, has been identified in human foetal membranes, where mycoplasma-derived lipopeptides can activate the NLRP7 inflammasome in amnion epithelial cells—a mechanism relevant to both physiological and pathological membrane rupture [22]. Together with the red- and orange-complex species described above, this broadens the set of maternal oral microorganisms potentially relevant to gestational outcomes.

Based on this evidence, we selected four salivary targets: (i) *P. gingivalis*, as the archetypal red-complex periodontopathogen with documented translocation to the placenta and amniotic fluid [15,18]; (ii) *F. nucleatum*, as the dominant orange-complex bridging species with the most direct molecular mechanism for haematogenous translocation to the fetoplacental unit [15]; (iii) *S. mutans*, as the principal cariogenic species and predictor of early-childhood caries through vertical transmission [19,21]; and (iv) *M. salivarium*, as a less-studied oral organism with a plausible mechanistic link to foetal membrane inflammation [22]. This panel spans the two dominant maternal oral disease processes—periodontal dysbiosis and dental caries—while including one emerging candidate with foetal membrane relevance.

Although the association between maternal oral microbial carriage and adverse pregnancy outcomes is well recognised, published studies vary widely in case definitions, sampling methodology, and population characteristics. Beyond direct gestational effects, maternal oral pathogen carriage at delivery has implications for child oral health: *S. mutans* is transmitted vertically from mother to infant during the perinatal window and is the primary determinant of early-childhood caries [21], while emerging evidence suggests that periodontopathogenic species such as *P. gingivalis* and *F. nucleatum* may also be horizontally acquired by neonates during early colonisation, potentially shaping the child’s developing oral microbiome [10]. To our knowledge, no study has previously characterised these four targets simultaneously in postpartum saliva in a Northeast Romanian obstetric cohort.

AIM: This exploratory study aimed to: (1) establish baseline salivary prevalence estimates for four oral pathogens—*P. gingivalis*, *S. mutans*, *F. nucleatum*, and *M. salivarium*—in a postpartum obstetric cohort in Northeast Romania using Real-Time PCR, and (2) describe co-detection patterns across clinical and demographic categories to inform the design of future confirmatory studies in this population.

## 2. Materials and Methods

### 2.1. Study Design and Patient Cohort

Between 1 December 2025 and 15 February 2026, we included 60 postpartum women hospitalised at the “Cuza-Vodă” Clinical Hospital of Obstetrics and Gynecology, Iași, Romania. Participants were recruited consecutively during the postpartum hospitalisation period, based on availability and willingness to participate, without matching or randomisation. No formal screening log was maintained; therefore, the exact number of eligible women who declined participation could not be quantified and should be considered a limitation. Because recruitment was hospital-based, consecutive, and dependent on participant availability, selection bias cannot be excluded. All participants were adults (18–49 years old), provided written informed consent, and completed a structured questionnaire regarding obstetric history, gynaecological background, oral hygiene habits, and oral health status.

The questionnaire included data regarding gestational age, parity, mode of delivery (vaginal delivery, caesarean section), premature rupture of membranes (defined as rupture of the foetal membranes prior to the onset of labour, irrespective of gestational age), history of dental caries, gingival bleeding during brushing, tooth mobility, smoking status, oral hygiene frequency, type of toothpaste used, toothbrush replacement frequency, time since the last dental visit, presence of natural teeth, dental implants, orthodontic appliances, Pap smear history, HPV testing, vaginal secretion microbiology, and associated systemic diseases (hypertension, diabetes mellitus, or other chronic conditions). Psychological conditions such as depression were not systematically assessed, representing a limitation given the potential influence of stress and immune modulation on the oral microbiome. Complete participant-level data for all variables collected are provided in Supplementary Table S1.

Two calibrated examiners performed oral cavity assessments focused on visible dental caries, gingival bleeding reported during brushing, tooth mobility, and general oral status. Before study initiation, the two examiners underwent joint training and calibration on the oral assessment criteria used in this study to standardise visual clinical evaluation. No standardised periodontal indices (such as probing depth or clinical attachment loss) were recorded; therefore, the study focused on descriptive oral health status rather than formal periodontal diagnosis.

Saliva samples were collected using CliniSwab LTS (Regione Monforte, Italy), swabs and placed in 1–3 mL transport medium for molecular bacterial testing. Samples were stored at 2–6 °C immediately after collection and processed the following morning.

Inclusion criteria were postpartum women aged ≥18 years who agreed to participate and had complete essential clinical data available. Exclusion criteria included: refusal to participate, incomplete clinical records, inability to provide informed consent, and receipt of antibiotic therapy within 30 days before sample collection, as antibiotic use may significantly alter oral microbiome composition. Use of other medications was recorded through the questionnaire but was not applied as an exclusion criterion.

This study was designed as an exploratory cross-sectional study. The primary objective was to generate baseline salivary prevalence data for four oral pathogens in a postpartum Romanian obstetric cohort, not to test predefined hypotheses or demonstrate clinical associations. The sample size of 60 was determined by the number of eligible women available during the study period and should be regarded as a convenience sample appropriate for an exploratory prevalence assessment. No formal a priori power calculation was performed, as this study was designed as an exploratory prevalence assessment rather than a hypothesis-testing trial; this represents an acknowledged limitation affecting statistical power for association analyses. The study did not include a non-pregnant control group. Enrolling a matched non-pregnant control arm within the same hospital setting within the study period was not logistically feasible given the convenience-based recruitment design and the limited availability of eligible participants; additionally, the primary aim was intra-cohort description rather than comparative inference. This omission prevents interpretation of whether the salivary prevalence rates observed here are elevated or reduced relative to a non-pregnant baseline and should be remedied in future confirmatory studies with a parallel control arm. The results are intended to inform the design and power calculations of future confirmatory studies.

### 2.2. DNA Extraction

Genomic DNA was extracted using the genesig^®^ Easy DNA/RNA Extraction Kit (Primerdesign Ltd., Chandler’s Ford, UK), a magnetic-bead-based kit validated by the manufacturer for use with biological matrices, including saliva. The kit was used within its specified shelf life, following the manufacturer’s protocol.

Briefly, 200 μL of each saliva sample was mixed with lysis buffer and internal extraction control, followed by sequential purification steps using magnetic separation and wash buffers (Tubes 3–6). After air drying, nucleic acids were eluted using 200 μL of elution buffer (Tube 7), and the final eluate volume used for downstream applications was 75 μL.

DNA extraction was performed the morning following sample collection to minimise degradation and preserve sample integrity.

### 2.3. Real-Time PCR Assay

Real-time PCR was performed using species-specific genesig^®^ Advanced kits (Primerdesign Ltd., Chandler’s Ford, UK), each commercially available and validated by the manufacturer for the corresponding target. Four oral bacterial species were assayed, selected for their relevance in periodontal dysbiosis, dental caries, and adverse pregnancy outcomes: *Porphyromonas gingivalis* (*fimA* I), *Streptococcus mutans* (glucosyltransferase-I), *Mycoplasma salivarium* (*rpoB*), and *Fusobacterium nucleatum* (*fus1*). All kits were used within their specified shelf life. *P. gingivalis* and *F. nucleatum* were selected as key periodontal pathogens frequently associated with periodontal inflammation and adverse pregnancy outcomes, including premature rupture of membranes and preterm birth. *S. mutans* was included as the principal cariogenic pathogen and a recognised target of vertical maternal–child oral microbial transmission. *M. salivarium* was selected as an emerging opportunistic species associated with oral dysbiosis, owing to its capacity to activate the NLRP7 inflammasome in amnion epithelial cells [22].

Each assay employed TaqMan^®^ hydrolysis-probe chemistry (5′ nuclease assay) with species-specific primers and probes validated by the manufacturer against >95% of reference sequences for each species in the NCBI nucleotide database. Each 20 µL reaction contained the lyophilised primer/probe mix (resuspended per manufacturer’s instructions), PrecisionPLUS 2× qPCR Master Mix, and 5 µL of extracted DNA template, made up with nuclease-free water to the final reaction volume. Thermal cycling was performed on a Stratagene Mx3005P qPCR system (Agilent Technologies, Santa Clara, CA, USA) under the following conditions: initial enzyme activation at 95 °C for 2 min (1 cycle), followed by 50 cycles of denaturation at 95 °C for 10 s and combined annealing/extension at 60 °C for 60 s, with fluorescence acquisition in the FAM channel during each annealing/extension step. Cycling was extended to 50 cycles, in line with manufacturer recommendations, to maximise detection of low-abundance taxa.

Each run included both positive and negative controls. The positive control consisted of the pathogen-specific Positive Control Template supplied within each genesig^®^ Advanced kit, while RNase/DNase-free water served as the no-template control (NTC). For assay validation, positive controls were required to amplify within the manufacturer-specified range of Ct 16–23, and NTCs were required to remain undetermined (no Ct) throughout 50 cycles; both criteria were met in every run prior to clinical sample analysis. Across all runs, observed Ct values for the positive controls remained within narrow lot-specific intervals (*P. gingivalis* 17.05–17.56; *S. mutans* 17.89–18.28; *M. salivarium* 17.84–21.04; *F. nucleatum* 21.09–21.19), indicating consistent inter-run reproducibility. Clinical samples showing detectable amplification within 50 cycles were classified as positive; samples without detectable amplification were recorded as “no Ct” and classified as negative. Ct values were retained for descriptive analysis as a semi-quantitative indicator of bacterial load. Sample-level Ct values for all 60 participants and the corresponding amplification plots are provided in Supplementary Materials (Table S2 and Figure S1).

No culture-independent sequencing approaches, such as 16S rRNA amplicon sequencing or shotgun metagenomics, were applied in this exploratory study. The targeted four-plex TaqMan-based panel was selected to provide quantitative, species-specific prevalence data for four clinically pre-specified organisms within the logistical and cost constraints of a single-centre convenience sample; whole-microbiome profiling is identified as a methodological priority for future confirmatory studies.

### 2.4. Statistical Analysis

Statistical analysis was performed using IBM SPSS Statistics version 27.0 (IBM Corp., Armonk, NY, USA).

Descriptive statistics were used for both categorical and continuous variables. Frequencies and percentages were calculated for categorical variables, including pathogen detection, patient characteristics, oral hygiene behaviours, and obstetric and gynaecological variables. Continuous variables such as age and Ct values were summarised using ranges and approximate median values.

Bivariate analyses were performed using Pearson’s chi-square test or Fisher’s exact test, as appropriate, to evaluate associations between bacterial detection and participant characteristics, including age group, gingival bleeding, dental caries, ruptured membranes, smoking status, and oral hygiene variables. Pearson’s chi-square test was used for variables with more than two categories. Fisher’s exact test was used for all 2 × 2 comparisons. A two-sided *p*-value < 0.05 was considered statistically significant. Given the universal detection of *Fusobacterium nucleatum* in the study cohort, comparative statistical analysis for this species was not applicable (N/A).

Given the exploratory nature of the study, analyses were intended to describe patterns of detection rather than to provide stable effect-size estimates for confirmatory inference; accordingly, no adjustment for multiple comparisons was applied, and all inferential analyses should be interpreted as hypothesis-generating. The absence of statistically significant associations should be interpreted in the context of limited statistical power rather than as evidence against established biological relationships.

### 2.5. Ethical Considerations

The study was conducted according to the principles of the Declaration of Helsinki and received approval from the Ethics Committee of the “Cuza Vodă” Clinical Hospital of Obstetrics and Gynecology, Iași, Romania (Approval No. 23, 23 October 2025), and from the Ethics Committee of the “Grigore T. Popa” University of Medicine and Pharmacy, Iași, Romania (Approval No. 711, 3 February 2026).

Recruitment took place exclusively after delivery, when participants were clinically stable; saliva collection is itself non-invasive. To address the risk of coercion or undue influence in this vulnerable population, the following safeguards applied: (i) consent was obtained by a research team member uninvolved in the participant’s clinical management; (ii) participants were informed verbally and in writing that declining or later withdrawing would not affect their own care, their newborn’s care, or their relationship with the hospital; (iii) consent was sought during the stable postpartum window rather than around acute clinical decisions and (iv) data and samples were anonymised by sequential participant code, with the linkage key held separately from the clinical team. Written informed consent was obtained from every participant after a briefing on the study aims, procedures, confidentiality, and right to withdraw. Strict confidentiality of patient information was maintained throughout the study.

## 3. Results

Given the exploratory nature of this study, the following results are presented as baseline prevalence estimates and descriptive distributions. No hypothesis-confirmatory inferences are drawn. All statistical comparisons are exploratory and should be interpreted in the context of limited statistical power.

### 3.1. Real-Time PCR Results

All Real-Time PCR experiments met the pre-defined quality control criteria. Negative controls (no-template controls), tested in duplicate for each pathogen, consistently produced no detectable amplification (no Ct), confirming the absence of contamination. Positive controls, also run in duplicate, yielded Ct values within the expected per-assay ranges: *P. gingivalis* 17.05–17.56; *S. mutans* 17.89–18.28; *M. salivarium* 17.84–21.04; *F. nucleatum* 21.09–21.19. These values confirm adequate assay sensitivity and reaction efficiency for each target.

Among positive clinical samples, high bacterial loads (Ct < 29, used here as a descriptive indicator) were identified in a subset of participants: three participants showed *P. gingivalis* Ct values below 29 (minimum Ct 28.04), and seven participants showed *S. mutans* Ct values below 29 (minimum Ct 27.12). These findings support detectable bacterial carriage at relatively higher DNA loads within this cohort. No *M. salivarium* sample reached this threshold, consistent with the higher median Ct of 36.64, indicating uniformly low bacterial loads for this species across the cohort. *F. nucleatum* showed the highest proportion of high-load cases (14/60, 23.3% with Ct < 29), consistent with its role as an abundant oral commensal. Detection frequencies and Ct value distributions are presented in Table 1.

Since *F. nucleatum* was detected in all 60 participants, co-detection analysis focused on the three variable pathogens: *P. gingivalis*, *S. mutans*, and *M. salivarium*. Only one participant (1.7%) carried none of these three organisms, being positive for *F. nucleatum* alone.

Nine participants (15.0%) tested positive for all three variable pathogens simultaneously (*S. mutans* + *M. salivarium* + *P. gingivalis*). Thirty-one participants (51.7%) carried two of the three variable pathogens: the most frequent two-pathogen combination was *S. mutans* + *M. salivarium*, detected in 18/60 participants (30.0%), followed by *M. salivarium* + *P. gingivalis* in 8/60 (13.3%) and *S. mutans* + *P. gingivalis* in 5/60 (8.3%). Nineteen participants (31.7%) carried only one of the three variable pathogens: *S. mutans* alone in 10 cases (16.7%), *M. salivarium* alone in 8 cases (13.3%), and *P. gingivalis* alone in 1 case (1.7%). Co-detection patterns are presented in Table 2.

These co-detection patterns suggest that in a postpartum hospital cohort, poly-microbial oral carriage is the rule rather than the exception. These patterns represent the primary descriptive contribution of this study, providing the first quantitative estimates of poly-microbial oral carriage in postpartum women in Northeast Romania.

### 3.2. Pathogen Detection by Sociodemographic, Obstetric, and Gynaecological Characteristics

Table 3 presents the distribution of pathogen detection according to sociodemographic, obstetric, and gynaecological characteristics. Fisher’s exact test or chi-square test was applied to all categorical comparisons; a significance threshold of *p* < 0.05 was used. No statistically significant associations were identified between any of the four pathogens and any sociodemographic or obstetric variable, consistent with the small sample size and the exploratory nature of this study. The findings reported below are therefore purely descriptive.

Age distribution. Participants ranged in age from 18 to 49 years (mean 30.6 years), with the largest proportion in the 23–27 age group. The wide age range reflects the inclusive eligibility criteria applied in this study rather than intentional age-based sampling, as all consenting adult postpartum women meeting the inclusion criteria were eligible for participation. Three of the four pathogens (*S. mutans*, *M. salivarium*, and *F. nucleatum*) were detected in every age stratum, whereas *P. gingivalis* was not detected in the 18–22 or 48–49 age groups (each *n* = 2). This most likely reflects the small subgroup sizes rather than a genuine age effect, as no statistically significant association was identified (chi-square, *p* = 0.186). Although age is recognised as an important determinant of oral microbiome composition, the limited sample size precluded reliable age-stratified analysis, and this should be addressed in larger confirmatory studies.

Educational attainment and socioeconomic status. The majority of participants had completed university-level education (61.7%) and were classified as having normal socioeconomic status (63.3%). Pathogen detection was distributed across all educational and socioeconomic subgroups with no markedly different pattern.

Obstetric characteristics. Most participants were primiparous (55.0%) and delivered at term (≥37 weeks of gestation; 78.3%). Caesarean section was the most frequent mode of delivery (60.0%). Premature rupture of membranes (PROM) was documented in 24 participants (40.0%). Among women with PROM, *P. gingivalis* was detected in 11/24 (45.8%) compared with 12/36 (33.3%) among women without PROM; this difference was not statistically significant (Fisher’s exact test, *p* = 0.419).

Gynaecological characteristics. Gynaecological variables were collected to characterise the overall reproductive health context of the cohort and were not primary study outcomes. No statistically significant associations were identified between any gynaecological variable and oral pathogen detection (all *p* > 0.05).

Systemic comorbidities. Comorbidities were present in 14/60 participants (23.3%), including arterial hypertension (*n* = 4), gastritis (*n* = 3), gestational diabetes (*n* = 2), and other conditions (*n* = 5). No statistically significant associations were identified between comorbidity status and pathogen detection; however, *P. gingivalis* showed a numerically higher detection rate in participants with any comorbidity compared with those without (57.1% vs. 32.6%; Fisher’s exact, *p* = 0.123), a trend consistent with the known immunomodulatory effects of systemic conditions on periodontal pathogen carriage [3] that warrants examination in larger studies. Of note, gestational diabetes was present in 2 participants (3.3%); the bidirectional relationship between dental caries and diabetes—including its gestational form—is well documented in the literature and represents an additional factor potentially relevant to the oral pathogen carriage patterns observed in this subgroup [23].

### 3.3. Pathogen Detection by Oral Hygiene Behaviours, Periodontal Indicators, and Dental Caries Status

Methodological note: No standardised periodontal clinical indices were recorded in this study. Probing depth, clinical attachment level, and formal bleeding-on-probing scores were not assessed; consequently, no clinical diagnosis of periodontitis was established in any participant. All references to periodontal indicators in this section reflect patient-reported symptoms confirmed on visual oral inspection only, and no inference regarding active periodontal disease is made.

Table 4 presents pathogen detection according to oral hygiene behaviours and clinical oral findings. No statistically significant associations were identified (all *p* > 0.05; full *p*-values in Table 4).

Dental caries. *S. mutans* was detected in 71.1% of participants with caries versus 66.7% without (Fisher’s exact, *p* = 0.754). *P. gingivalis* was detected in 35.6% with caries versus 46.7% without (Fisher’s exact, *p* = 0.544). Although *S. mutans* is the primary aetiological agent of dental caries, no statistically significant difference in detection frequency was identified between caries-positive and caries-negative participants, likely reflecting limited statistical power rather than a true absence of association.

Gingival bleeding during brushing. Gingival bleeding was reported by 44/60 (73.3%) participants. Salivary detection frequencies in bleeding-positive versus bleeding-negative participants were *P. gingivalis* 38.6% vs. 37.5% (Fisher’s exact, *p* = 1.000); *S. mutans* 72.7% vs. 62.5% (Fisher’s exact, *p* = 0.529); *M. salivarium* 70.5% vs. 75.0% (Fisher’s exact, *p* = 1.000). No significant differences were identified.

Tooth mobility. *P. gingivalis* was detected in 42.1% of participants with tooth mobility versus 36.6% without (Fisher’s exact, *p* = 0.778). *M. salivarium* detection was numerically higher in participants with mobility (84.2%) than without (65.9%), though this did not reach significance (Fisher’s exact, *p* = 0.219).

Smoking. *M. salivarium* detection was numerically higher in smokers (87.5%) versus non-smokers (65.9%), though this did not reach significance (Fisher’s exact, *p* = 0.120). This trend is biologically plausible given the established effect of tobacco smoke on oral microbiome composition and warrants examination in larger studies.

## 4. Discussion

Because no standardised periodontal examination was performed, the present findings concern salivary pathogen carriage and self-reported or visually assessed oral signs rather than clinically confirmed periodontal disease. This exploratory study provides the first baseline salivary prevalence estimates for four clinically relevant oral pathogens in postpartum women in Northeast Romania, generated using quantitative Real-Time PCR with commercially validated TaqMan-based assays. As an exploratory prevalence study, it was not designed to confirm associations between pathogen detection and clinical outcomes; the absence of statistically significant associations in this cohort is therefore expected, given the sample size, and should not be interpreted as evidence against the well-established biological links between these organisms and oral or gestational pathology. The principal scientific contributions are: (1) baseline prevalence data for four oral pathogens in a population not previously characterised in this way; (2) the first description of poly-microbial co-detection patterns in this setting; and (3) empirical data supporting power calculations for future confirmatory studies. Saliva samples were collected immediately after delivery at a tertiary obstetric referral centre; therefore, the cohort likely overrepresents higher-risk pregnancies compared with the general obstetric population, and findings should not be generalised beyond a hospital-based setting.

### 4.1. Rationale for the Selected Bacterial Panel

*Porphyromonas gingivalis* is a keystone member of the red complex, the group of periodontal pathogens most strongly associated with destructive periodontitis; its capacity to dysregulate the host immune response and to translocate haematogenously to the placenta makes it the most clinically prioritised target in periodontal-obstetric research [18]. *Streptococcus mutans* is the primary etiological agent of dental caries and a well-validated salivary biomarker for caries risk; its inclusion was motivated by the high caries burden expected in a pregnant population and by its established vertical transmission to the neonate [21]. *Fusobacterium nucleatum* was included as a structural bridging organism central to oral biofilm architecture and as a species repeatedly identified in adverse placental findings [24,25]. *Mycoplasma salivarium*, while less studied than the preceding three, was selected based on emerging evidence for its presence in foetal membranes and its capacity to activate the NLRP7 inflammasome pathway in amnion epithelial cells [22]. Together, this panel covers the major dimensions of caries risk, periodontal pathogen carriage, biofilm organisation, and emerging inflammatory pathways.

### 4.2. Interpreting Bacterial Detection: Colonisation Versus Disease

Real-Time PCR with TaqMan probes detects bacterial DNA with high sensitivity but does not differentiate between active infection, subclinical colonisation, or residual non-viable DNA. The detection of a pathogen in saliva therefore does not, by itself, confirm disease or clinical pathology. This caveat applies to all four organisms studied and is especially important when interpreting the universal or near-universal salivary detection frequencies observed for some species.

Saliva was chosen as the sampling matrix because of its non-invasive collection procedure, its feasibility in a postpartum hospital setting, and its established utility for detecting oral pathogens at a population level. These practical advantages come at a cost in specificity: whole saliva dilutes organisms concentrated in periodontal pockets or interdental spaces. The salivary detection prevalence reported here reflects oral carriage across the entire oral cavity rather than site-specific pathogenic colonisation. Future studies would benefit from supplementing salivary sampling with subgingival plaque, which concentrates periodontal pathogens more reliably and is the reference matrix in most periodontal microbiological research.

The detection of *Fusobacterium nucleatum* in all participants most likely reflects its role as a commensal and structural component of the normal oral biofilm [24]. While *F. nucleatum* has been identified in placental and amniotic tissues of women experiencing preterm delivery, and its FadA adhesin-mediated disruption of endothelial barrier integrity represents a plausible translocation mechanism [25,26], such pathogenicity cannot be inferred from salivary DNA detection alone. Its 100% prevalence also renders it analytically uninformative for association analyses. The clinical relevance of salivary *F. nucleatum* detection in obstetric populations has been supported by culture-based studies. A culture-based investigation in postnatal mothers reported statistically significant increases in *F. nucleatum* colony-forming units in the unstimulated saliva of mothers who delivered preterm compared with those who delivered at term, suggesting that higher oral bacterial load may be clinically meaningful even when detected in saliva rather than placental tissue [27]. A previous PCR-based investigation detected *F. nucleatum* by PCR in seven chorionic tissue samples from high-risk pregnant women, while all oral samples—including those from normal-risk women—tested positive, a pattern closely paralleling the 100% salivary detection observed in our cohort [28]. Together, these findings reinforce the interpretation that universal salivary detection of *F. nucleatum* reflects normal oral carriage, while translocation to chorionic or placental tissue represents a secondary pathological event that salivary sampling alone cannot capture.

The high salivary prevalence of *Mycoplasma salivarium* (71.7%) warrants cautious interpretation. To our knowledge, this represents one of the first quantitative estimates of this organism in an obstetric population using Real-Time PCR and warrants targeted investigation given its proposed role in foetal membrane inflammasome activation [22]. Comparison with existing studies is constrained by the scarcity of research targeting this pathogen specifically in obstetric cohorts. The prevalence reported here should be regarded as hypothesis-generating. Prior to emerging evidence of its role in foetal membrane biology, *M. salivarium* was characterised primarily as an opportunistic pathogen in immunocompromised hosts. Pyrosequencing of salivary microbiota in hospitalised patients with AIDS and different periodontal statuses has identified *M. salivarium* as part of the opportunistic microbiome in necrotic periodontitis, alongside *Capnocytophaga* spp. and *Neisseria elongata* [29]. While our cohort consisted of immunocompetent postpartum women, this earlier work establishes that *M. salivarium* can colonise the oral cavity at detectable levels under conditions of host immune modulation—a mechanism that may also be relevant to the pregnancy–postpartum continuum.

Co-detection analysis revealed that 9 participants (15%) carried all three variable pathogens simultaneously (*S. mutans, M. salivarium* and *P. gingivalis*), and a further 31 (51.7%) carried two of the three. The most frequent two-pathogen combination was *S. mutans* + *M. salivarium* (18/60, 30.0%), consistent with their shared niche in supragingival biofilm. These co-detection patterns suggest that poly-microbial oral carriage is the rule rather than the exception in this postpartum hospital cohort, which has implications for the design of future targeted interventions.

The use of four separate single-plex PCR runs rather than a validated multiplex assay is a limitation affecting laboratory efficiency. A multiplex approach, if commercially available, would be more suitable for future studies of this scale and would reduce inter-run variability.

### 4.3. Oral Bacteria and Pregnancy Outcomes

The association between oral pathogens and adverse pregnancy outcomes, including preterm birth, low birth weight, preeclampsia, and PROM, has been increasingly reported in the literature, though mechanistic certainty remains elusive [15,16,18]. Among the 24 participants (40.0%) with documented PROM, *P. gingivalis* was detected in 11/24 (45.8%), compared with 12/36 (33.3%) among women without PROM; this difference was not statistically significant (Fisher’s exact test, *p* = 0.419). This directional difference is consistent with the broader literature suggesting that *P. gingivalis* virulence factors may influence uterine inflammatory signalling [18,30]; however, no causal inference can be drawn from cross-sectional detection-based data, and because samples were collected postpartum rather than during gestation, any association with pregnancy outcomes is retrospective and indirect.

The high salivary prevalence of *S. mutans* has implications beyond maternal oral health. Vertical mother-to-child transmission of *S. mutans* is a well-established primary route of early-childhood caries acquisition [21]. Detectable maternal salivary carriage during the perinatal period therefore represents a potentially modifiable risk factor for caries in the offspring, supporting consideration of maternal oral health management within prenatal and perinatal care pathways. Direct evidence for *S. mutans* translocation beyond the oral cavity during pregnancy has been reported, with this organism detected by real-time PCR in 69.2% of amniotic fluid samples from women with uncomplicated term deliveries, 65% showing simultaneous salivary and amniotic-fluid detection, and a positive association observed with infrequent dental attendance [31]. The 70.0% salivary prevalence of *S. mutans* observed in the present cohort is consistent with those maternal carriage levels and supports the biological plausibility of oral-to-amniotic translocation.

### 4.4. Clinical Oral Health Findings

The high prevalence of dental caries (75.0%) and gingival bleeding (73.3%) in this cohort warrants comment from a descriptive clinical perspective. Gingival bleeding during toothbrushing is not equivalent to a diagnosis of periodontitis. Because participants were sampled immediately postpartum, reported gingival bleeding likely reflects oral changes that developed during pregnancy, most commonly pregnancy gingivitis—an exaggerated vascular inflammatory response driven by elevated progesterone and oestrogen levels that is generally reversible postpartum [6]. Without clinical probing data, the observed bleeding cannot be attributed to destructive periodontitis. This distinction matters when situating these findings within the periodontal-pregnancy literature, where the biological plausibility of systemic effects has been more consistently linked to periodontitis than to reversible gingivitis. No participant underwent a formal periodontal examination; therefore, our findings relate to self-reported or visually assessed oral signs rather than clinically diagnosed periodontitis.

*S. mutans* was detected in 71.1% of participants with dental caries versus 66.7% without (*p* = 0.754), and *P. gingivalis* was detected in 38.6% of participants with gingival bleeding versus 37.5% without (*p* = 1.000). The absence of statistically significant associations is consistent with the small sample size and the exploratory design; it should not be interpreted as evidence against the well-established biological links between these organisms and their respective diseases.

The high rate of dental caries despite relatively favourable self-reported hygiene practices is consistent with the physiological cariogenic changes documented during pregnancy, which may help explain the postpartum oral findings observed here, including reduced salivary calcium and phosphate and increasing *S. mutans* counts in the later trimesters [20]. The disconnect between reported hygiene behaviour and observed oral findings reflects a well-known limitation of questionnaire-based oral health data.

These findings are situated within a Romanian context where oral health literacy and dental attendance during and around pregnancy remain suboptimal [32]. The 75.0% prevalence of dental caries and 73.3% rate of gingival bleeding observed here are consistent with patterns documented in comparable European obstetric cohorts, reinforcing the case for integrating oral health education into antenatal programmes [33,34]. Internationally, comparable caries burdens have been reported in obstetric cohorts from diverse settings. A national Colombian sample of pregnant women documented a 59% caries prevalence, with 89.9% having experienced caries at some point, and identified socioeconomic determinants as key modifying factors [35]. Further evidence indicates that low oral health literacy modifies the relationship between socioeconomic status and untreated dental caries in pregnant women, with those combining low income and low oral health literacy showing the highest caries burden [36]. Although direct comparison is limited by differences in case definition and population characteristics, the consistently high caries prevalence across these settings reinforces the need for structured oral health education as a component of antenatal care, regardless of geographic context.

### 4.5. Behavioural and Sociodemographic Factors

A gap between perceived and practised oral health was evident in this cohort. Although most participants reported oral hygiene as important, nearly half had not attended a dental appointment in over a year, and most used cosmetic rather than therapeutic toothpaste. This pattern likely reflects systemic barriers to dental care access before and during pregnancy, including concerns about foetal safety, cost, and the absence of integrated dental referral pathways in antenatal services, which may have influenced the postpartum findings observed in this cohort. A substantial proportion of women brushed only once a day (in the morning), a practice that leaves the oral cavity vulnerable to the nocturnal maturation of biofilms [5].

A numerically higher detection rate of *M. salivarium* was observed in smokers compared with non-smokers (87.5% vs. 65.9%), though this did not reach statistical significance (*p* = 0.120). This trend is biologically plausible given the established effect of tobacco smoke on oral microbiome composition and warrants examination in larger studies with sufficient power to detect modest effect sizes.

### 4.6. Comparison with Previous Studies: Methodological Considerations

Comparison of our salivary detection prevalence with that reported in previous studies requires careful attention to methodological differences. Studies reporting higher *P. gingivalis* prevalence than observed here typically used subgingival plaque or interdental microbiota sampling—compartments that concentrate periodontal pathogens more reliably than whole saliva [37]. Population differences also apply: our cohort consisted of postpartum women in a hospital setting, whereas most comparative studies sampled women during active gestation. The classification of PCR results as positive also varies across studies. Geographic region, sample size, primer targets, and PCR platform all contribute additional variability that precludes direct numerical comparison.

By “multidisciplinary approach,” we refer to coordinated care between obstetric teams and dental practitioners, which may include oral health screening, referral to dental services, and patient education within routine antenatal care pathways. A randomised trial has demonstrated that daily use of calibrated interdental brushes reduced gingival bleeding from 56% to 12% by 8 months of pregnancy [38]. Given the 73.3% rate of gingival bleeding in our cohort, this intervention may be evaluated in future prospective prenatal oral-care studies, although it was not assessed directly in the present postpartum cohort.

From a methodological perspective, the use of quantitative PCR for salivary pathogen detection in this study is consistent with broader advances in molecular periodontal diagnostics. Recent reviews of molecular periodontal diagnostics note that advances in PCR-based methods have made quantitative assessment of specific periodontal pathogens increasingly accessible and have resulted in a range of commercially validated assays suitable for clinical use [39]. A CFU-based qPCR quantification method has been developed and validated for six oral bacteria—including *P. gingivalis*, *F. nucleatum* and *S. mutans*—demonstrating that such approaches provide highly accurate and reproducible quantitation of oral pathogenic bacteria [40]. In the Romanian context, multiplex PCR has been applied to identify periodontal pathogens in patients with periodontitis, reporting *Treponema denticola* and *P. gingivalis* to be the most frequent targets, with molecular detection combined with minimum inhibitory concentration-based antibiogram analysis to guide therapeutic decisions [41]. Complementary work has shown that real-time PCR-based pathogen detection can be complemented by host mRNA analysis in patients positive for *P. gingivalis*, *Aggregatibacter actinomycetemcomitans* and *Tannerella forsythia*, broadening the diagnostic potential of molecular assays in periodontal research [42]. These methodological precedents support the validity of the qPCR approach used in the present study and situate our findings within an established and growing tradition of molecular oral microbiological research in this region.

### 4.7. Limitations

In summary, and consolidating the methodological caveats already integrated into the preceding subsections, the principal limitations of this study are: a small convenience sample (*n* = 60) recruited consecutively at a single tertiary obstetric hospital, which restricts statistical power and external generalisability; the absence of a non-pregnant control group and of multivariate analysis, which together preclude controlled comparative inference; the use of whole saliva rather than subgingival plaque, which lowers specificity for site-specific periodontal pathogens; the absence of standardised periodontal indices, which precludes formal periodontal diagnosis; the cross-sectional postpartum collection window, which prevents inference about microbial dynamics during gestation; and the universal detection of *F. nucleatum* precluded association analysis for this organism. Additional caveats include the absence of a formal refusal log, no systematic assessment of psychological conditions such as depression, and the absence of blinding during clinical or laboratory assessment.

### 4.8. Implications for Future Study Design

The prevalence data generated in this exploratory study permit prospective power calculations for future confirmatory research. Using the observed *P. gingivalis* prevalence of 38.3% and the directional difference in detection between women with and without PROM (45.8% vs. 33.3%), future confirmatory studies would likely require a substantially larger sample, in the range of 200–250 participants per group, depending on the final design and target effect size. For *S. mutans* and dental caries (71.1% vs. 66.7%), the smaller observed difference would require a substantially larger sample.

These estimates suggest that confirmatory association studies in this population should incorporate a non-pregnant control arm, apply standardised periodontal clinical indices (probing depth, clinical attachment level, bleeding-on-probing), and include longitudinal sampling across pregnancy trimesters. Extending the pathogen panel to include *Treponema denticola*, *Tannerella forsythia*, *Prevotella* spp., and *Capnocytophaga* spp. would provide a more comprehensive picture of oral dysbiosis [43]. The present study provides the baseline prevalence framework upon which such designs can be built.

## 5. Conclusions

This exploratory study is the first in Northeast Romania to characterise salivary carriage of *P. gingivalis*, *S. mutans*, *M. salivarium*, and *F. nucleatum* simultaneously in postpartum women using quantitative Real-Time PCR. *Fusobacterium nucleatum* was detected in all participants, consistent with its role as a ubiquitous oral commensal. Among the three variable pathogens, *S. mutans* and *M. salivarium* showed the highest prevalence (70.0% and 71.7% respectively), and poly-microbial carriage was common, with 15.0% of participants carrying all three variable pathogens simultaneously and the most frequent two-pathogen combination being *S. mutans* + *M. salivarium* (30.0%).

No statistically significant associations were identified between pathogen detection and clinical or obstetric variables, a finding consistent with the limited statistical power of a small convenience sample rather than evidence against established biological relationships. These results reflect the descriptive scope of this study and do not support causal inferences.

These baseline prevalence estimates and co-detection patterns serve as an empirical foundation for future prospective studies with larger sample sizes, standardised periodontal assessment, non-pregnant control groups, and longitudinal sampling across pregnancy. The observed 71.7% prevalence of *M. salivarium* supports further investigation in larger confirmatory studies, particularly in view of its proposed mechanistic relevance to foetal membrane inflammation. Such studies are needed to establish whether the oral pathogen carriage rates documented here translate into clinically measurable associations with gestational outcomes in the Romanian obstetric population.

## Figures and Tables

**Table 1:** Salivary detection frequency and Ct value distribution for the four oral pathogens (*n* = 60).

Pathogen	Detection *n*/N (%)	Ct Range (Min–Max)	Median Ct	Descriptive Note
*P. gingivalis*	23/60 (38.3%)	28.04–38.82	33.09	Baseline prevalence 38.3%; informs future power calculations
*S. mutans*	42/60 (70.0%)	27.12–46.45	33.78	Baseline prevalence 70.0%; most frequent variable pathogen
*M. salivarium*	43/60 (71.7%)	31.45–40.23	36.64	Baseline prevalence 71.7%; novel estimate for this population
*F. nucleatum*	60/60 (100.0%)	25.13–35.43	30.77	100% detection; excluded from association analyses

Ct: Cycle threshold. Lower values indicate higher bacterial DNA load. Any detectable amplification was classified as positive. The 100% detection of *F. nucleatum* is consistent with its role as a ubiquitous commensal of the normal oral microbiome and does not, by itself, indicate active infection or disease.

**Table 2:** Co-detection patterns for variable oral pathogens (*n* = 60).

Variable Pathogen Combination	*n*	%
All three variable pathogens (*S. mutans* + *M. salivarium* + *P. gingivalis*)	9	15.0%
*S. mutans* + *M. salivarium* only	18	30.0%
*M. salivarium* + *P. gingivalis* only	8	13.3%
*S. mutans* + *P. gingivalis* only	5	8.3%
*S. mutans* only	10	16.7%
*M. salivarium* only	8	13.3%
*P. gingivalis* only	1	1.7%
*F. nucleatum* only (no variable pathogens)	1	1.7%
Total	60	100%

Note: *F. nucleatum* was detected in all 60 participants and is therefore excluded from combination counts. The “*F. nucleatum* only” category refers to the single participant with no other variable pathogen detected. Percentages are rounded to one decimal place; therefore, component percentages may not sum exactly to the total.

**Table 3:** Distribution of oral bacterial detection according to sociodemographic, obstetric, and gynaecological characteristics (*n* = 60).

Characteristic	Total *n* (%)	*S. mutans n* (%)	*P. gingivalis n* (%)	*M. salivarium n* (%)	*F. nucleatum n* (%)	*p*-Value †
Distribution by age group (years)						*S.m.*: 0.536; *P.g.*: 0.186; *M.s.*: 0.910; *F.n.*: N/A
18–22	2 (3.3%)	2 (3.3%)	0 (0.0%)	1 (1.7%)	2 (3.3%)	
23–27	21 (35.0%)	13 (21.7%)	11 (18.3%)	15 (25.0%)	21 (35.0%)	
28–32	16 (26.7%)	13 (21.7%)	3 (5.0%)	11 (18.3%)	16 (26.7%)	
33–37	15 (25.0%)	9 (15.0%)	7 (11.7%)	12 (20.0%)	15 (25.0%)	
38–42	4 (6.7%)	3 (5.0%)	2 (3.3%)	3 (5.0%)	4 (6.7%)	
48–49	2 (3.3%)	2 (3.3%)	0 (0.0%)	1 (1.7%)	2 (3.3%)	
Distribution by educational attainment						*S.m.*: 0.648; *P.g.*: 0.220; *M.s.*: 0.661; *F.n.*: N/A
Primary education	2 (3.3%)	1 (1.7%)	1 (1.7%)	2 (3.3%)	2 (3.3%)	
High school	21 (35.0%)	16 (26.7%)	11 (18.3%)	15 (25.0%)	21 (35.0%)	
University	37 (61.7%)	25 (41.7%)	11 (18.3%)	26 (43.3%)	37 (61.7%)	
Distribution by marital status						*S.m.*: 0.686; *P.g.*: 1.000; *M.s.*: 1.000; *F.n.*: N/A
Unmarried	8 (13.3%)	5 (8.3%)	3 (5.0%)	6 (10.0%)	8 (13.3%)	
Married	52 (86.7%)	37 (61.7%)	20 (33.3%)	37 (61.7%)	52 (86.7%)	
Distribution by area of residence						*S.m.*: 0.773; *P.g.*: 0.281; *M.s.*: 0.777; *F.n.*: N/A
Rural	23 (38.3%)	17 (28.3%)	11 (18.3%)	16 (26.7%)	23 (38.3%)	
Urban	37 (61.7%)	25 (41.7%)	12 (20.0%)	27 (45.0%)	37 (61.7%)	
Distribution by socioeconomic status						*S.m.*: 0.703; *P.g.*: 0.825; *M.s.*: 0.260; *F.n.*: N/A
Low	38 (63.3%)	28 (46.7%)	14 (23.3%)	25 (41.7%)	38 (63.3%)	
Medium	16 (26.7%)	10 (16.7%)	6 (10.0%)	14 (23.3%)	16 (26.7%)	
High	6 (10.0%)	4 (6.7%)	3 (5.0%)	4 (6.7%)	6 (10.0%)	
Gestational age						*S.m.*: 0.328; *P.g.*: 0.801; *M.s.*: 0.787; *F.n.*: N/A
<32 weeks	2 (3.3%)	2 (3.3%)	1 (1.7%)	1 (1.7%)	2 (3.3%)	
32–36 weeks	11 (18.3%)	6 (10.0%)	5 (8.3%)	8 (13.3%)	11 (18.3%)	
≥37 weeks	47 (78.3%)	34 (56.7%)	17 (28.3%)	34 (56.7%)	47 (78.3%)	
Previous pregnancies						*S.m.*: 0.591; *P.g.*: 0.080; *M.s.*: 0.449; *F.n.*: N/A
0	33 (55.0%)	24 (40.0%)	9 (15.0%)	23 (38.3%)	33 (55.0%)	
1	18 (30.0%)	13 (21.7%)	8 (13.3%)	12 (20.0%)	18 (30.0%)	
≥ 2	9 (15.0%)	5 (8.3%)	6 (10.0%)	8 (13.3%)	9 (15.0%)	
Mode of delivery						*S.m.*: 0.573; *P.g.*: 1.000; *M.s.*: 1.000; *F.n.*: N/A
Vaginal	24 (40.0%)	18 (30.0%)	9 (15.0%)	17 (28.3%)	24 (40.0%)	
Caesarean section	36 (60.0%)	24 (40.0%)	14 (23.3%)	26 (43.3%)	36 (60.0%)	
Ruptured membranes						*S.m.*: 1.000; *P.g.*: 0.419; *M.s.*: 1.000; *F.n.*: N/A
No	36 (60.0%)	25 (41.7%)	12 (20.0%)	26 (43.3%)	36 (60.0%)	
Yes	24 (40.0%)	17 (28.3%)	11 (18.3%)	17 (28.3%)	24 (40.0%)	
History of abnormal Pap smear results						*S.m.*: 0.547; *P.g.*: 1.000; *M.s.*: 1.000; *F.n.*: N/A
Normal	57 (95.0%)	39 (65.0%)	22 (36.7%)	41 (68.3%)	57 (95.0%)	
Abnormal (ASC-US/LSIL/HSIL)	3 (5.0%)	3 (5.0%)	1 (1.7%)	2 (3.3%)	3 (5.0%)	
History of HPV testing						*S.m.*: 0.300; *P.g.*: 1.000; *M.s.*: 1.000; *F.n.*: N/A
Negative	59 (98.3%)	42 (70.0%)	23 (38.3%)	42 (70.0%)	59 (98.3%)	
Positive	1 (1.7%)	0 (0.0%)	0 (0.0%)	1 (1.7%)	1 (1.7%)	
Vaginal secretion—specific microorganisms						*S.m.*: 0.910; *P.g.*: 0.421; *M.s.*: 0.904; *F.n.*: N/A
Negative	43 (71.7%)	30 (50.0%)	15 (25.0%)	31 (51.7%)	43 (71.7%)	
*E. coli*	4 (6.7%)	3 (5.0%)	1 (1.7%)	3 (5.0%)	4 (6.7%)	
*Candida* spp.	12 (20.0%)	8 (13.3%)	6 (10.0%)	8 (13.3%)	12 (20.0%)	
*E. coli* + *Candida* spp.	1 (1.7%)	1 (1.7%)	1 (1.7%)	1 (1.7%)	1 (1.7%)	
Systemic comorbidities						*S.m.*: 0.520; *P.g.*: 0.123; *M.s.*: 0.737; *F.n.*: N/A
None	46 (76.7%)	31 (51.7%)	15 (25.0%)	32 (53.3%)	46 (76.7%)	
Any comorbidity ^1^	14 (23.3%)	11 (18.3%)	8 (13.3%)	11 (18.3%)	14 (23.3%)	

Note: † *p*-values were obtained using Pearson’s chi-square test for variables with more than two categories. Fisher’s exact test was used for all 2 × 2 comparisons. *F. nucleatum* was detected in 100% of samples, so no statistical test was applicable (N/A). All comparisons remained non-significant (*p* > 0.05). No women aged 43–47 years were enrolled in this study. ^1^ Systemic comorbidities included: arterial hypertension (*n* = 4), gastritis (*n* = 3), gestational diabetes (*n* = 2), autoimmune thyroiditis (*n* = 1), gestational hypertension (*n* = 1), rheumatoid arthritis (*n* = 1), fibromyalgia (*n* = 1), and type I diabetes mellitus (*n* = 1). Individual subgroup sizes were insufficient for stratified statistical analysis; therefore, participants were dichotomised as ‘None’ versus ‘Any comorbidity’ for descriptive purposes only.

**Table 4:** Association between oral bacterial detection and oral hygiene behaviours, periodontal indicators, and dental caries status (*n* = 60).

Characteristic	Total *n* (%)	*S. mutans n* (%)	*P. gingivalis n* (%)	*M. salivarium n* (%)	*F. nucleatum n* (%)	*p*-Value †
Oral hygiene frequency						*S.m.*: 0.281; *P.g.*: 0.376; *M.s.*: 0.784; *F.n.*: N/A
Once daily	21 (35.0%)	17 (28.3%)	6 (10.0%)	14 (23.3%)	21 (35.0%)	
Twice daily	34 (56.7%)	21 (35.0%)	14 (23.3%)	25 (41.7%)	34 (56.7%)	
After each meal	5 (8.3%)	4 (6.7%)	3 (5.0%)	4 (6.7%)	5 (8.3%)	
Perceived importance of oral hygiene						*S.m.*: 1.000; *P.g.*: 1.000; *M.s.*: 1.000; *F.n.*: N/A
No	4 (6.7%)	3 (5.0%)	1 (1.7%)	3 (5.0%)	4 (6.7%)	
Yes	56 (93.3%)	39 (65.0%)	22 (36.7%)	40 (66.7%)	56 (93.3%)	
Type of toothpaste						*S.m.*: 0.631; *P.g.*: 1.000; *M.s.*: 0.616; *F.n.*: N/A
Therapeutic	5 (8.3%)	3 (5.0%)	2 (3.3%)	3 (5.0%)	5 (8.3%)	
Cosmetic	55 (91.7%)	39 (65.0%)	21 (35.0%)	40 (66.7%)	55 (91.7%)	
Toothbrush replacement frequency						*S.m.*: 0.263; *P.g.*: 0.973; *M.s.*: 0.739; *F.n.*: N/A
Monthly	12 (20.0%)	9 (15.0%)	5 (8.3%)	8 (13.3%)	12 (20.0%)	
Every 3 months	41 (68.3%)	26 (43.3%)	15 (25.0%)	31 (51.7%)	41 (68.3%)	
Every 6 months	5 (8.3%)	5 (8.3%)	2 (3.3%)	3 (5.0%)	5 (8.3%)	
Once a year	2 (3.3%)	2 (3.3%)	1 (1.7%)	1 (1.7%)	2 (3.3%)	
Time since last dental visit						*S.m.*: 0.693; *P.g.*: 0.384; *M.s.*: 0.726; *F.n.*: N/A
<1 month	4 (6.7%)	3 (5.0%)	0 (0.0%)	2 (3.3%)	4 (6.7%)	
1–3 months	3 (5.0%)	3 (5.0%)	2 (3.3%)	2 (3.3%)	3 (5.0%)	
3–6 months	9 (15.0%)	5 (8.3%)	3 (5.0%)	6 (10.0%)	9 (15.0%)	
6–12 months	17 (28.3%)	12 (20.0%)	8 (13.3%)	14 (23.3%)	17 (28.3%)	
>1 year	27 (45.0%)	19 (31.7%)	10 (16.7%)	19 (31.7%)	27 (45.0%)	
Smoking status						*S.m.*: 0.207; *P.g.*: 0.765; *M.s.*: 0.120; *F.n.*: N/A
Non-smoker	44 (73.3%)	33 (55.0%)	16 (26.7%)	29 (48.3%)	44 (73.3%)	
Smoker	16 (26.7%)	9 (15.0%)	7 (11.7%)	14 (23.3%)	16 (26.7%)	
Dental caries status						*S.m.*: 0.754; *P.g.*: 0.544; *M.s.*: 1.000; *F.n.*: N/A
No	15 (25.0%)	10 (16.7%)	7 (11.7%)	11 (18.3%)	15 (25.0%)	
Yes	45 (75.0%)	32 (53.3%)	16 (26.7%)	32 (53.3%)	45 (75.0%)	
Gingival bleeding during brushing						*S.m.*: 0.529; *P.g.*: 1.000; *M.s.*: 1.000; *F.n.*: N/A
No	16 (26.7%)	10 (16.7%)	6 (10.0%)	12 (20.0%)	16 (26.7%)	
Yes	44 (73.3%)	32 (53.3%)	17 (28.3%)	31 (51.7%)	44 (73.3%)	
Tooth mobility						*S.m.*: 0.227; *P.g.*: 0.778; *M.s.*: 0.219; *F.n.*: N/A
No	41 (68.3%)	31 (51.7%)	15 (25.0%)	27 (45.0%)	41 (68.3%)	
Yes	19 (31.7%)	11 (18.3%)	8 (13.3%)	16 (26.7%)	19 (31.7%)	
Dental status: natural teeth present						*S.m.*: 1.000; *P.g.*: 1.000; *M.s.*: 1.000; *F.n.*: N/A
No	1 (1.7%)	1 (1.7%)	0 (0.0%)	1 (1.7%)	1 (1.7%)	
Yes	59 (98.3%)	41 (68.3%)	23 (38.3%)	42 (70.0%)	59 (98.3%)	
Dental implant status						*S.m.*: 0.478; *P.g.*: 0.306; *M.s.*: 0.265; *F.n.*: N/A
No	49 (81.7%)	33 (55.0%)	17 (28.3%)	37 (61.7%)	49 (81.7%)	
Yes	11 (18.3%)	9 (15.0%)	6 (10.0%)	6 (10.0%)	11 (18.3%)	
Presence of orthodontic appliance						*S.m.*: 0.348; *P.g.*: 0.777; *M.s.*: 0.209; *F.n.*: N/A
No	43 (71.7%)	32 (53.3%)	16 (26.7%)	33 (55.0%)	43 (71.7%)	
Yes	17 (28.3%)	10 (16.7%)	7 (11.7%)	10 (16.7%)	17 (28.3%)	
Oral hygiene frequency						*S.m.*: 0.281; *P.g.*: 0.376; *M.s.*: 0.784; *F.n.*: N/A
Once daily	21 (35.0%)	17 (28.3%)	6 (10.0%)	14 (23.3%)	21 (35.0%)	
Twice daily	34 (56.7%)	21 (35.0%)	14 (23.3%)	25 (41.7%)	34 (56.7%)	
After each meal	5 (8.3%)	4 (6.7%)	3 (5.0%)	4 (6.7%)	5 (8.3%)	
Perceived importance of oral hygiene						*S.m.*: 1.000; *P.g.*: 1.000; *M.s.*: 1.000; *F.n.*: N/A
No	4 (6.7%)	3 (5.0%)	1 (1.7%)	3 (5.0%)	4 (6.7%)	
Yes	56 (93.3%)	39 (65.0%)	22 (36.7%)	40 (66.7%)	56 (93.3%)	
Type of toothpaste						*S.m.*: 0.631; *P.g.*: 1.000; *M.s.*: 0.616; *F.n.*: N/A
Therapeutic	5 (8.3%)	3 (5.0%)	2 (3.3%)	3 (5.0%)	5 (8.3%)	
Cosmetic	55 (91.7%)	39 (65.0%)	21 (35.0%)	40 (66.7%)	55 (91.7%)	
Toothbrush replacement frequency						*S.m.*: 0.263; *P.g.*: 0.973; *M.s.*: 0.739; *F.n.*: N/A
Monthly	12 (20.0%)	9 (15.0%)	5 (8.3%)	8 (13.3%)	12 (20.0%)	
Every 3 months	41 (68.3%)	26 (43.3%)	15 (25.0%)	31 (51.7%)	41 (68.3%)	
Every 6 months	5 (8.3%)	5 (8.3%)	2 (3.3%)	3 (5.0%)	5 (8.3%)	
Once a year	2 (3.3%)	2 (3.3%)	1 (1.7%)	1 (1.7%)	2 (3.3%)	
Time since last dental visit						*S.m.*: 0.693; *P.g.*: 0.384; *M.s.*: 0.726; *F.n.*: N/A
<1 month	4 (6.7%)	3 (5.0%)	0 (0.0%)	2 (3.3%)	4 (6.7%)	
1–3 months	3 (5.0%)	3 (5.0%)	2 (3.3%)	2 (3.3%)	3 (5.0%)	
3–6 months	9 (15.0%)	5 (8.3%)	3 (5.0%)	6 (10.0%)	9 (15.0%)	
6–12 months	17 (28.3%)	12 (20.0%)	8 (13.3%)	14 (23.3%)	17 (28.3%)	
>1 year	27 (45.0%)	19 (31.7%)	10 (16.7%)	19 (31.7%)	27 (45.0%)	
Smoking status						*S.m.*: 0.207; *P.g.*: 0.765; *M.s.*: 0.120; *F.n.*: N/A
Non-smoker	44 (73.3%)	33 (55.0%)	16 (26.7%)	29 (48.3%)	44 (73.3%)	
Smoker	16 (26.7%)	9 (15.0%)	7 (11.7%)	14 (23.3%)	16 (26.7%)	
Dental caries status						*S.m.*: 0.754; *P.g.*: 0.544; *M.s.*: 1.000; *F.n.*: N/A
No	15 (25.0%)	10 (16.7%)	7 (11.7%)	11 (18.3%)	15 (25.0%)	
Yes	45 (75.0%)	32 (53.3%)	16 (26.7%)	32 (53.3%)	45 (75.0%)	
Gingival bleeding during brushing						*S.m.*: 0.529; *P.g.*: 1.000; *M.s.*: 1.000; *F.n.*: N/A
No	16 (26.7%)	10 (16.7%)	6 (10.0%)	12 (20.0%)	16 (26.7%)	
Yes	44 (73.3%)	32 (53.3%)	17 (28.3%)	31 (51.7%)	44 (73.3%)	
Tooth mobility						*S.m.*: 0.227; *P.g.*: 0.778; *M.s.*: 0.219; *F.n.*: N/A
No	41 (68.3%)	31 (51.7%)	15 (25.0%)	27 (45.0%)	41 (68.3%)	
Yes	19 (31.7%)	11 (18.3%)	8 (13.3%)	16 (26.7%)	19 (31.7%)	
Dental status: natural teeth present						*S.m.*: 1.000; *P.g.*: 1.000; *M.s.*: 1.000; *F.n.*: N/A
No	1 (1.7%)	1 (1.7%)	0 (0.0%)	1 (1.7%)	1 (1.7%)	
Yes	59 (98.3%)	41 (68.3%)	23 (38.3%)	42 (70.0%)	59 (98.3%)	
Dental implant status						*S.m.*: 0.478; *P.g.*: 0.306; *M.s.*: 0.265; *F.n.*: N/A
No	49 (81.7%)	33 (55.0%)	17 (28.3%)	37 (61.7%)	49 (81.7%)	
Yes	11 (18.3%)	9 (15.0%)	6 (10.0%)	6 (10.0%)	11 (18.3%)	
Presence of orthodontic appliance						*S.m.*: 0.348; *P.g.*: 0.777; *M.s.*: 0.209; *F.n.*: N/A
No	43 (71.7%)	32 (53.3%)	16 (26.7%)	33 (55.0%)	43 (71.7%)	
Yes	17 (28.3%)	10 (16.7%)	7 (11.7%)	10 (16.7%)	17 (28.3%)	

Note: † *p*-values were obtained using Pearson’s chi-square test for variables with more than two categories. Fisher’s exact test was used for all 2 × 2 comparisons. *F. nucleatum* was detected in 100% of samples, so no statistical test was applicable (N/A). All comparisons remained non-significant (*p* > 0.05).

## Data Availability

All data supporting the findings of this study, including the complete sample-level cycle threshold (Ct) dataset for all 60 participants and the corresponding amplification plots, are provided in the Supplementary Materials (Table S2 and Figure S1). Anonymised participant-level clinical, obstetric, gynaecological, oral health, and microbiological data for all 60 participants are provided in Supplementary Table S2.

## Supplementary Materials

The following supporting information can be downloaded at https://www.mdpi.com/article/10.3390/pathogens15050507/s1, Figure S1: Amplification plots for samples 1–60; Table S1: Individual participant-level clinical, obstetric, gynaecological, oral health, and microbiological data for all 60 postpartum women (*n* = 60). Table S2: Cycle threshold (Ct) values for oral bacterial species detected by real-time PCR (*n* = 60).
