# Supplementary material for: Salivary Prevalence of Four Oral Pathogens in Postpartum Women in Northeast Romania: An Exploratory Cross-Sectional Study
Source: Pathogens. 2026 May 8;15(5):507. doi: 10.3390/pathogens15050507 (PMC13209267; doi:10.3390/pathogens15050507)
Supplement: Supplementary file 1 [file pathogens-15-00507-s001.zip › pathogens-4282071 Supplementary figures.pdf]

Table S1. Individual participant-level clinical, obstetric, gynaecological, oral health, and microbiological data for all 60 postpartum women (*n* = 60).

ID	Sociodemographic characteristics							Obstetric characteristics				Oral health and hygiene											Gynaecological characteristics			Microbiological results							
**Participant ID**	**Age (years)**	**Area of residence**	**Marital status**	**Educational attainment**	**Socioeconomic status**	**Smoking status**	**Systemic comorbidities**	**Gestational age (weeks)**	**Previous pregnancies**	**Mode of delivery**	**Ruptured membranes**	**Dental caries**	**Gingival bleeding**	**Tooth mobility**	**Natural teeth**	**Dental implants**	**Orthodontic appliance**	**Oral hygiene frequency**	**Importance of oral hygiene**	**Type of toothpaste**	**Toothbrush replacement**	**Time since last dental visit**	**Pap smear result**	**HPV testing**	**Vaginal secretion**	*S. mutans detection*	*S. mutans Ct*	*F. nucleatum detection*	*F. nucleatum Ct*	*M. salivarium detection*	*M. salivarium Ct*	*P. gingivalis detection*	*P. gingivalis Ct*
1	27	Urban	Married	High school	Normal	No	No	38	1	Cesarean section	Yes	Yes	Yes	Yes	Yes	No	No	Twice daily	Yes	Cosmetic	Monthly	Less than 3 months	No	Negative	No	Detected	32.31	Detected	34.13	Not detected	No Ct	Detected	32.72
2	34	Urban	Married	University	High	No	No	38	0	Cesarean section	Yes	Yes	No	No	Yes	No	No	Twice daily	Yes	Cosmetic	Every 3 months	Less than 6 months	No	Negative	No	Not detected	No Ct	Detected	31.28	Detected	38.62	Not detected	No Ct
3	31	Urban	Married	High school	Medium	No	No	38	2	Cesarean section	No	Yes	No	Yes	Yes	No	No	Twice daily	Yes	Cosmetic	Every 3 months	Between 6 months and 1 year	No	Negative	No	Detected	38.28	Detected	26.54	Detected	36.72	Not detected	No Ct
4	29	Urban	Married	University	Normal	No	No	38	0	Cesarean section	No	Yes	Yes	No	Yes	Yes	Yes	After each meal	Yes	Therapeutic	Every 3 months	Less than 6 months	No	Negative	No	Detected	29.98	Detected	31.48	Not detected	No Ct	Not detected	No Ct
5	23	Rural	Unmarried	High school	Normal	Yes	No	40	3	Vaginal	No	Yes	Yes	No	Yes	No	No	Twice daily	Yes	Cosmetic	Monthly	More than 1 year	No	Negative	No	Not detected	No Ct	Detected	25.46	Detected	36.68	Detected	33.09
6	29	Rural	Married	High school	Normal	No	No	38	1	Cesarean section	Yes	Yes	No	No	Yes	No	No	Twice daily	Yes	Cosmetic	Monthly	More than 1 year	No	Negative	No	Detected	29.00	Detected	29.31	Not detected	No Ct	Not detected	No Ct
7	27	Urban	Married	University	Medium	Yes	No	38	0	Cesarean section	No	No	Yes	No	Yes	No	No	Twice daily	Yes	Cosmetic	Every 3 months	More than 1 year	No	Negative	No	Detected	27.68	Detected	28.78	Detected	34.82	Not detected	No Ct
8	30	Urban	Married	High school	Normal	No	No	38	0	Vaginal	No	Yes	Yes	No	Yes	No	No	Twice daily	Yes	Cosmetic	Every 6 months	More than 1 year	ASC-US	Negative	No	Detected	46.45	Detected	27.58	Detected	34.37	Not detected	No Ct
9	35	Urban	Married	University	Medium	No	Gastritis	38	1	Cesarean section	Yes	Yes	Yes	Yes	Yes	No	No	Once daily	Yes	Therapeutic	Every 3 months	More than 1 year	No	Negative	No	Not detected	No Ct	Detected	31.37	Detected	35.72	Not detected	No Ct
10	29	Urban	Married	University	Medium	No	No	38	2	Vaginal	Yes	Yes	Yes	Yes	Yes	No	Yes	Once daily	Yes	Cosmetic	Every 3 months	Less than 1 month	No	Negative	No	Detected	36.24	Detected	33.31	Detected	37.50	Not detected	No Ct
11	49	Urban	Unmarried	University	High	No	No	39	1	Cesarean section	No	Yes	Yes	No	Yes	Yes	No	Once daily	Yes	Cosmetic	Every 3 months	Less than 1 month	No	Negative	Escherichia coli	Detected	33.48	Detected	29.44	Not detected	No Ct	Not detected	No Ct
12	42	Urban	Married	University	Medium	No	No	39	2	Cesarean section	No	Yes	Yes	No	Yes	No	No	Once daily	Yes	Cosmetic	Every 6 months	More than 1 year	No	Negative	Escherichia coli	Detected	35.58	Detected	31.89	Detected	37.98	Not detected	No Ct
13	30	Urban	Married	High school	Medium	No	No	35	1	Vaginal	Yes	Yes	Yes	Yes	Yes	No	No	Once daily	Yes	Cosmetic	Every 3 months	More than 1 year	No	Negative	No	Not detected	No Ct	Detected	33.07	Detected	35.26	Not detected	No Ct
14	25	Urban	Married	High school	Medium	No	No	40	0	Vaginal	No	Yes	No	No	Yes	No	No	Twice daily	Yes	Cosmetic	Monthly	Between 6 months and 1 year	No	Negative	No	Not detected	No Ct	Detected	25.13	Detected	33.83	Not detected	No Ct
15	27	Rural	Married	High school	Normal	No	Gastritis	38	0	Vaginal	No	Yes	Yes	No	Yes	No	No	Once daily	Yes	Cosmetic	Monthly	More than 1 year	No	Negative	No	Detected	36.23	Detected	32.74	Detected	36.37	Not detected	No Ct
16	27	Rural	Married	University	Normal	No	No	38	0	Cesarean section	No	Yes	No	No	Yes	No	No	After each meal	Yes	Cosmetic	Every 3 months	More than 1 year	No	Negative	No	Not detected	No Ct	Detected	32.50	Detected	39.62	Not detected	No Ct
17	27	Rural	Married	University	Normal	Yes	No	40	0	Cesarean section	Yes	Yes	Yes	No	Yes	No	No	Twice daily	Yes	Cosmetic	Every 3 months	More than 1 year	No	Negative	No	Detected	34.96	Detected	26.62	Detected	31.45	Not detected	No Ct
18	26	Urban	Married	University	Normal	No	No	36	0	Cesarean section	Yes	Yes	Yes	Yes	Yes	No	Yes	Twice daily	Yes	Cosmetic	Every 3 months	Between 6 months and 1 year	No	Negative	No	Not detected	No Ct	Detected	33.29	Detected	32.78	Not detected	No Ct
19	23	Rural	Married	High school	Normal	No	No	38	1	Vaginal	Yes	Yes	Yes	Yes	Yes	No	No	Once daily	No	Cosmetic	Once a year	More than 1 year	No	Negative	No	Detected	36.99	Detected	32.81	Not detected	No Ct	Detected	35.43
20	25	Rural	Married	High school	Normal	Yes	Arterial hypertension	37	0	Cesarean section	No	No	No	No	Yes	No	No	Twice daily	Yes	Cosmetic	Every 3 months	More than 1 year	No	Negative	Escherichia coli	Not detected	No Ct	Detected	28.85	Detected	35.24	Detected	32.29
21	33	Urban	Married	University	Medium	Yes	No	38	0	Vaginal	No	No	Yes	No	Yes	No	Yes	Twice daily	Yes	Therapeutic	Every 3 months	Less than 1 month	No	Positive	No	Not detected	No Ct	Detected	29.78	Detected	36.65	Not detected	No Ct
22	40	Urban	Married	University	Medium	No	No	36	0	Vaginal	No	Yes	Yes	Yes	Yes	No	No	Twice daily	Yes	Cosmetic	Every 3 months	More than 1 year	No	Negative	Candida spp.	Not detected	No Ct	Detected	29.88	Detected	39.51	Detected	37.36
23	29	Rural	Married	University	Medium	No	No	29	0	Vaginal	No	Yes	No	No	Yes	No	No	Twice daily	Yes	Cosmetic	Every 3 months	Between 6 months and 1 year	No	Negative	No	Detected	30.62	Detected	29.20	Not detected	No Ct	Detected	28.68
24	26	Rural	Married	University	Medium	No	No	38	0	Vaginal	No	No	Yes	No	Yes	No	Yes	Twice daily	Yes	Cosmetic	Every 3 months	Less than 1 month	No	Negative	No	Detected	34.94	Detected	30.33	Not detected	No Ct	Not detected	No Ct
25	24	Urban	Married	High school	Normal	No	Arterial hypertension	37	0	Cesarean section	No	No	Yes	Yes	Yes	No	No	After each meal	Yes	Cosmetic	Every 3 months	More than 1 year	No	Negative	No	Detected	36.64	Detected	33.99	Detected	37.72	Detected	33.49
26	37	Urban	Married	University	Normal	Yes	Autoimmune thyroiditis	38	1	Vaginal	Yes	Yes	Yes	Yes	Yes	No	Yes	Twice daily	Yes	Cosmetic	Every 3 months	Less than 6 months	No	Negative	No	Detected	35.23	Detected	31.12	Detected	40.23	Not detected	No Ct
27	32	Urban	Married	University	Normal	No	No	38	1	Vaginal	Yes	Yes	Yes	No	Yes	No	Yes	Twice daily	Yes	Cosmetic	Monthly	Between 6 months and 1 year	No	Negative	No	Detected	37.43	Detected	32.80	Not detected	No Ct	Not detected	No Ct
28	29	Urban	Married	University	Normal	No	No	39	1	Vaginal	Yes	Yes	Yes	No	Yes	Yes	Yes	Twice daily	Yes	Cosmetic	Every 3 months	Less than 6 months	No	Negative	No	Not detected	No Ct	Detected	32.86	Detected	35.93	Detected	32.84
29	35	Rural	Married	High school	Normal	No	Rheumatoid arthritis	37	1	Cesarean section	No	Yes	Yes	No	Yes	No	No	Once daily	Yes	Therapeutic	Monthly	More than 1 year	ASC-US	Negative	No	Detected	32.86	Detected	28.41	Not detected	No Ct	Detected	37.09
30	36	Urban	Married	University	Medium	No	No	39	5	Cesarean section	No	Yes	No	No	Yes	No	No	Twice daily	Yes	Cosmetic	Every 3 months	Less than 3 months	No	Negative	Escherichia coli/Candida spp.	Detected	39.10	Detected	28.72	Detected	34.44	Detected	34.30

ID	Sociodemographic characteristics							Obstetric characteristics				Oral health and hygiene										Gynaecological characteristics				Microbiological results							
Participant ID	Age (years)	Area of residence	Marital status	Educational attainment	Socioeconomic status	Smoking status	Systemic comorbidities	Gestational age (weeks)	Previous pregnancies	Mode of delivery	Ruptured membranes	Dental caries	Gingival bleeding	Tooth mobility	Natural teeth	Dental implants	Orthodontic appliance	Oral hygiene frequency	Importance of oral hygiene	Type of toothpaste	Toothbrush replacement	Time since last dental visit	Pap smear result	HPV testing	Vaginal secretion	*S. mutans detection*	*S. mutans Ct*	*F. nucleatum detection*	*F. nucleatum Ct*	*M. salivarium detection*	*M. salivarium Ct*	*P. gingivalis detection*	*P. gingivalis Ct*
31	40	Urban	Unmarried	University	High	Yes	Fibromyalgia	36	1	Vaginal	Yes	No	Yes	No	Yes	Yes	No	After each meal	Yes	Therapeutic	Every 3 months	More than 1 year	No	Negative	Candida spp.	Detected	32.89	Detected	30.20	Detected	36.66	Detected	31.38
32	30	Rural	Married	High school	Normal	No	No	39	0	Vaginal	Yes	No	No	No	Yes	No	No	Once daily	No	Cosmetic	Every 3 months	Less than 6 months	No	Negative	Candida spp.	Detected	28.20	Detected	31.79	Detected	33.28	Not detected	No Ct
33	35	Urban	Married	University	High	Yes	Arterial hypertension	38	2	Cesarean section	Yes	Yes	Yes	No	Yes	Yes	Yes	After each meal	Yes	Cosmetic	Every 3 months	Less than 6 months	No	Negative	Candida spp.	Detected	31.31	Detected	29.72	Detected	38.10	Detected	38.00
34	26	Rural	Married	University	Medium	No	No	37	2	Cesarean section	Yes	Yes	Yes	Yes	Yes	No	Yes	Twice daily	Yes	Cosmetic	Monthly	More than 1 year	No	Negative	Candida spp.	Not detected	No Ct	Detected	28.56	Detected	37.64	Detected	28.37
35	35	Urban	Married	University	Normal	No	No	38	2	Cesarean section	No	No	Yes	No	Yes	Yes	Yes	Twice daily	Yes	Cosmetic	Every 3 months	Less than 6 months	No	Negative	Candida spp.	Not detected	No Ct	Detected	32.26	Not detected	No Ct	Detected	38.82
36	18	Urban	Unmarried	Primary education	Normal	Yes	No	37	0	Cesarean section	No	Yes	Yes	No	Yes	No	No	Once daily	No	Cosmetic	Once a year	More than 1 year	No	Negative	Candida spp.	Detected	28.82	Detected	29.42	Detected	38.70	Not detected	No Ct
37	37	Urban	Married	High school	Medium	Yes	No	35	0	Cesarean section	Yes	No	No	Yes	Yes	No	Yes	Twice daily	Yes	Cosmetic	Every 3 months	Between 6 months and 1 year	No	Negative	No	Detected	39.42	Detected	35.43	Detected	36.04	Detected	38.22
38	37	Urban	Married	University	Normal	Yes	No	38	0	Cesarean section	No	Yes	Yes	Yes	Yes	Yes	No	Twice daily	Yes	Cosmetic	Monthly	Between 6 months and 1 year	No	Negative	No	Detected	27.57	Detected	32.66	Detected	37.18	Not detected	No Ct
39	35	Urban	Married	University	High	No	Gestational diabetes	37	1	Cesarean section	Yes	No	No	No	Yes	No	No	Twice daily	Yes	Cosmetic	Every 3 months	Between 6 months and 1 year	No	Negative	Candida spp.	Not detected	No Ct	Detected	32.61	Detected	36.57	Detected	33.24
40	23	Rural	Married	High school	Medium	No	Gestational hypertension	37	1	Cesarean section	No	Yes	Yes	No	Yes	Yes	Yes	Twice daily	Yes	Cosmetic	Every 6 months	Between 6 months and 1 year	No	Negative	No	Detected	27.12	Detected	29.84	Detected	36.64	Detected	28.04
41	36	Urban	Married	University	Normal	No	No	37	1	Cesarean section	No	No	Yes	No	Yes	No	No	Twice daily	Yes	Cosmetic	Every 3 months	Less than 3 months	No	Negative	No	Detected	27.87	Detected	29.47	Detected	32.61	Not detected	No Ct
42	32	Urban	Married	High school	Medium	No	No	40	0	Vaginal	Yes	Yes	Yes	No	Yes	No	No	Twice daily	Yes	Cosmetic	Monthly	More than 1 year	No	Negative	No	Detected	32.36	Detected	30.62	Detected	35.26	Not detected	No Ct
43	33	Urban	Married	University	Normal	Yes	No	39	1	Cesarean section	No	Yes	Yes	Yes	Yes	No	No	Once daily	No	Cosmetic	Every 3 months	Between 6 months and 1 year	No	Negative	No	Not detected	No Ct	Detected	30.91	Detected	37.89	Not detected	No Ct
44	24	Rural	Married	High school	Normal	No	Arterial hypertension	40	0	Vaginal	Yes	Yes	Yes	No	Yes	Yes	No	Once daily	Yes	Cosmetic	Every 3 months	Between 6 months and 1 year	No	Negative	No	Detected	31.66	Detected	27.00	Detected	37.74	Detected	31.73
45	22	Rural	Married	High school	Normal	Yes	Type I diabetes	39	0	Vaginal	Yes	Yes	Yes	No	Yes	No	Yes	Twice daily	Yes	Cosmetic	Every 3 months	Between 6 months and 1 year	No	Negative	No	Detected	32.13	Detected	29.56	Not detected	No Ct	Not detected	No Ct
46	24	Rural	Married	University	Normal	No	No	39	0	Cesarean section	No	Yes	Yes	No	Yes	No	No	Twice daily	Yes	Cosmetic	Every 3 months	More than 1 year	No	Negative	Candida spp.	Detected	33.69	Detected	34.83	Not detected	No Ct	Not detected	No Ct
47	41	Urban	Married	University	High	No	No	36	0	Cesarean section	No	Yes	No	No	Yes	Yes	No	Once daily	Yes	Cosmetic	Every 6 months	More than 1 year	No	Negative	No	Detected	39.28	Detected	33.93	Not detected	No Ct	Not detected	No Ct
48	28	Rural	Married	University	Normal	No	Gastritis	36	0	Cesarean section	No	Yes	Yes	Yes	Yes	No	No	Once daily	Yes	Cosmetic	Every 3 months	Less than 6 months	No	Negative	No	Detected	37.46	Detected	32.94	Detected	37.75	Not detected	No Ct
49	28	Rural	Married	University	Normal	No	No	38	1	Vaginal	No	No	No	No	Yes	No	No	Twice daily	Yes	Cosmetic	Every 3 months	Between 6 months and 1 year	No	Negative	No	Detected	31.97	Detected	29.46	Detected	33.63	Not detected	No Ct
50	25	Urban	Married	University	Normal	No	No	38	0	Vaginal	Yes	Yes	No	No	Yes	No	Yes	Once daily	Yes	Cosmetic	Every 3 months	More than 1 year	No	Negative	Candida spp.	Detected	37.75	Detected	34.51	Not detected	No Ct	Not detected	No Ct
51	32	Rural	Married	University	Normal	No	No	40	1	Vaginal	Yes	No	Yes	No	Yes	No	No	Twice daily	Yes	Cosmetic	Every 3 months	Between 6 months and 1 year	No	Negative	No	Detected	35.45	Detected	30.07	Detected	39.05	Detected	32.87
52	23	Urban	Married	High school	Normal	No	No	37	0	Vaginal	Yes	Yes	Yes	No	Yes	No	No	Once daily	Yes	Cosmetic	Every 6 months	More than 1 year	No	Negative	No	Detected	35.13	Detected	28.75	Not detected	No Ct	Detected	37.34
53	34	Urban	Married	University	Normal	No	No	35	0	Cesarean section	No	Yes	Yes	No	Yes	No	No	Once daily	Yes	Cosmetic	Monthly	Between 6 months and 1 year	No	Negative	No	Detected	33.86	Detected	31.87	Detected	34.13	Detected	37.27
54	31	Rural	Married	University	Normal	No	No	37	0	Cesarean section	No	Yes	Yes	Yes	Yes	No	No	Twice daily	Yes	Cosmetic	Every 3 months	Between 6 months and 1 year	No	Negative	Candida spp.	Detected	32.91	Detected	31.00	Detected	33.80	Not detected	No Ct
55	27	Rural	Married	High school	Normal	Yes	No	39	0	Cesarean section	No	Yes	Yes	Yes	Yes	No	No	Twice daily	Yes	Cosmetic	Every 3 months	Between 6 months and 1 year	No	Negative	No	Not detected	No Ct	Detected	31.97	Detected	35.90	Detected	32.27
56	35	Urban	Married	University	Normal	No	Gestational diabetes	34	0	Cesarean section	No	No	Yes	Yes	Yes	Yes	No	Once daily	Yes	Cosmetic	Every 3 months	More than 1 year	No	Negative	Candida spp.	Detected	37.82	Detected	32.24	Not detected	No Ct	Not detected	No Ct
57	23	Rural	Unmarried	Primary education	Normal	Yes	No	34	2	Cesarean section	No	Yes	No	Yes	Yes	No	Yes	Once daily	Yes	Cosmetic	Every 3 months	More than 1 year	No	Negative	No	Not detected	No Ct	Detected	27.63	Detected	36.89	Detected	31.49
58	27	Rural	Unmarried	High school	Normal	No	No	38	0	Cesarean section	No	Yes	No	No	No	No	No	Once daily	Yes	Cosmetic	Every 3 months	More than 1 year	No	Negative	Escherichia coli	Detected	36.05	Detected	31.34	Detected	39.14	Not detected	No Ct
59	29	Urban	Unmarried	University	Normal	Yes	No	35	0	Cesarean section	No	No	Yes	No	Yes	No	Yes	Twice daily	Yes	Cosmetic	Every 3 months	Less than 6 months	No	Negative	No	Not detected	No Ct	Detected	29.40	Not detected	No Ct	Not detected	No Ct
60	48	Urban	Unmarried	University	Normal	No	No	27	1	Vaginal	No	Yes	Yes	No	Yes	No	No	Once daily	Yes	Cosmetic	Monthly	More than 1 year	ASC-US	Negative	No	Detected	27.40	Detected	27.91	Detected	35.25	Not detected	No Ct

Abbreviations: Ct, cycle threshold; PROM, premature rupture of membranes; ASC-US, atypical squamous cells of undetermined significance; LSIL, low-grade squamous intraepithelial lesion; HSIL, high-grade squamous intraepithelial lesion; HPV, human papillomavirus. Detected = positive amplification; Not detected = no detectable amplification (No Ct). All participants are identified by sequential number only; no personally identifiable information is included. Ct values reported as recorded by the MX3005P Stratagene thermocycler; 'No Ct' denotes absence of detectable

amplification.

Table S2. Cycle threshold (Ct) values for oral bacterial species detected by real-time PCR (*n* = 60).

Sample	*S. mutans Ct*	*F. nucleatum Ct*	*M. salivarium Ct*	*P. gingivalis Ct*
1	32.31	34.13	No Ct *	32.72
2	No Ct	31.28	38.62	No Ct
3	38.28	26.54	36.72	No Ct
4	29.98	31.48	No Ct	No Ct
5	No Ct	25.46	36.68	33.09
6	29.00	29.31	No Ct	No Ct
7	27.68	28.78	34.82	No Ct
8	46.45	27.58	34.37	No Ct
9	No Ct	31.37	35.72	No Ct
10	36.24	33.31	37.50	No Ct
11	33.48	29.44	No Ct	No Ct
12	35.58	31.89	37.98	No Ct
13	No Ct	33.07	35.26	No Ct
14	No Ct	25.13	33.83	No Ct
15	36.23	32.74	36.37	No Ct
16	No Ct	32.50	39.62	No Ct
17	34.96	26.62	31.45	No Ct
18	No Ct	33.29	32.78	No Ct
19	36.99	32.81	No Ct	35.43
20	No Ct	28.85	35.24	32.29
21	No Ct	29.78	36.65	No Ct
22	No Ct	29.88	39.51	37.36
23	30.62	29.20	No Ct	28.68
24	34.94	30.33	No Ct	No Ct
25	36.64	33.99	37.72	33.49
26	35.23	31.12	40.23	No Ct
27	37.43	32.80	No Ct	No Ct
28	No Ct	32.86	35.93	32.84
29	32.86	28.41	No Ct	37.09
30	39.10	28.72	34.44	34.30
31	32.89	30.20	36.66	31.38
32	28.20	31.79	33.28	No Ct
33	31.31	29.72	38.10	38.00
34	No Ct	28.56	37.64	28.37
35	No Ct	32.26	No Ct	38.82
36	28.82	29.42	38.70	No Ct
37	39.42	35.43	36.04	38.22
38	27.57	32.66	37.18	No Ct
39	No Ct	32.61	36.57	33.24
40	27.12	29.84	36.64	28.04
41	27.87	29.47	32.61	No Ct
42	32.36	30.62	35.26	No Ct
43	No Ct	30.91	37.89	No Ct
44	31.66	27.00	37.74	31.73
45	32.13	29.56	No Ct	No Ct
46	33.69	34.83	No Ct	No Ct

Sample	*S. mutans Ct*	*F. nucleatum Ct*	*M. salivarium Ct*	*P. gingivalis Ct*
47	39.28	33.93	No Ct	No Ct
48	37.46	32.94	37.75	No Ct
49	31.97	29.46	33.63	No Ct
50	37.75	34.51	No Ct	No Ct
51	35.45	30.07	39.05	32.87
52	35.13	28.75	No Ct	37.34
53	33.86	31.87	34.13	37.27
54	32.91	31.00	33.80	No Ct
55	No Ct	31.97	35.90	32.27
56	37.82	32.24	No Ct	No Ct
57	No Ct	27.63	36.89	31.49
58	36.05	31.34	39.14	No Ct
59	No Ct	29.40	No Ct	No Ct
60	27.40	27.91	35.25	No Ct

* No Ct = no detectable amplification within the predefined cycle threshold.

Streptococcus mutans

For *Streptococcus mutans*, amplification was evaluated using FAM-labeled assays. All no-template controls (NTCs) showed no amplification signal (No Ct), confirming the absence of contamination. Positive controls consistently amplified with Ct values ranging from 17.89 to 18.28, indicating optimal assay performance and reaction efficiency.

Among clinical samples, detected cases showed Ct values predominantly in the late amplification range, with Ct values spanning approximately 27 to 46 cycles, reflecting variable bacterial load among positive samples. Samples without detectable amplification were classified as negative.

Fusobacterium nucleatum

For *Fusobacterium nucleatum*, all NTC wells remained negative (No Ct), confirming assay specificity. Positive control reactions amplified consistently with Ct values of 21.09 and 21.19, demonstrating robust assay sensitivity.

Clinical samples showed widespread amplification, with Ct values distributed mainly between 25 and 35 cycles, consistent with the high prevalence of *F. nucleatum* detected in the study population.

Mycoplasma salivarium

Detection of *Mycoplasma salivarium* was characterised by a combination of positive and negative amplification results. All NTCs showed no amplification (No Ct), excluding non-specific signals or contamination.

Positive controls amplified reproducibly, with Ct values ranging from 17.84 to 21.04, confirming adequate assay sensitivity. In clinical samples, detected cases exhibited high Ct values, generally between 33 and 40 cycles, indicating low bacterial loads.

Porphyromonas gingivalis

For *Porphyromonas gingivalis*, all negative controls (NTCs) remained amplification-free (No Ct). Positive controls consistently generated amplification signals with Ct values between 17.05 and 17.56, demonstrating excellent assay performance.

Positive clinical samples showed Ct values primarily between 28 and 38 cycles, reflecting moderate bacterial loads and a heterogeneous distribution among participants.

Figure S1. Amplification plots for samples 1–60.

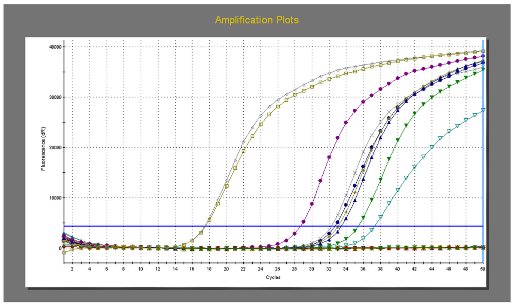

P. gingivalis samples 1 – 25

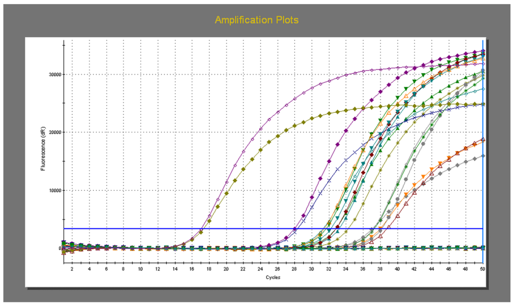

P. gingivalis samples 26 – 60

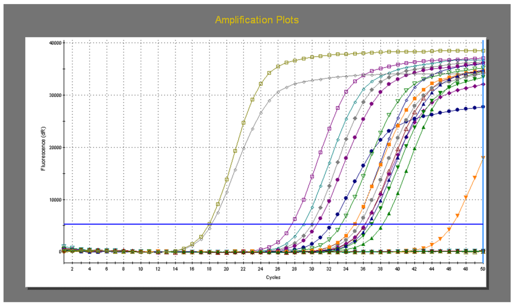

S. mutans samples 1 – 25

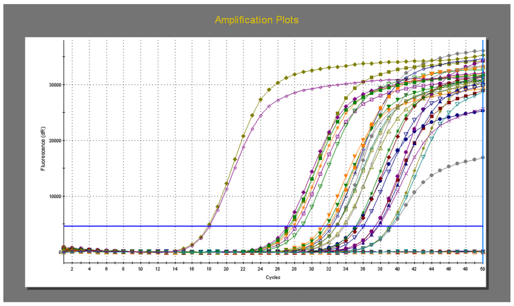

S. mutans samples 26 – 60

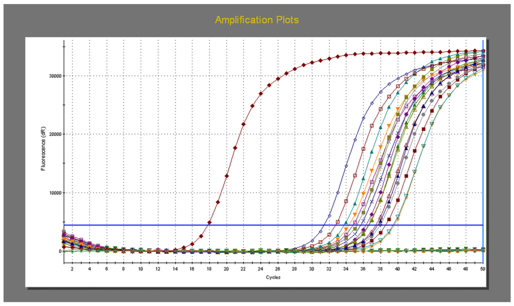

M. salivarium samples 1 – 25

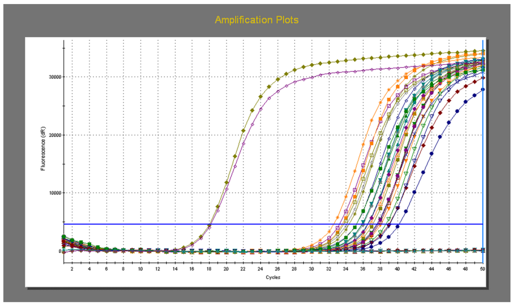

M. salivarium samples 26 – 60

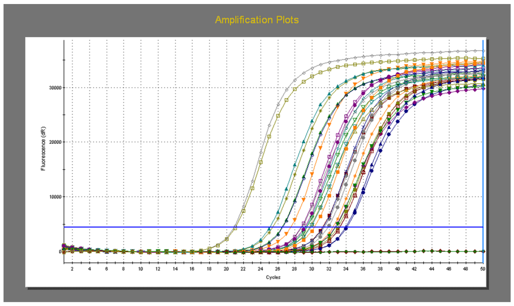

F. nucleatum samples 1 – 25

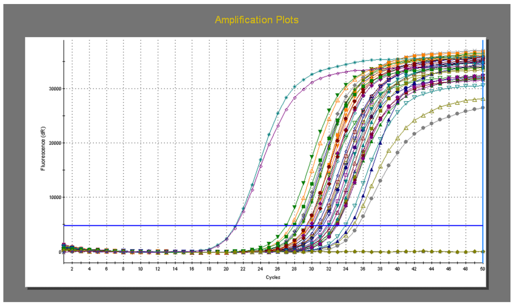

F. nucleatum samples 26 – 60
